# Murine Models Provide New Insights Into Pathogenesis of Chronic Graft-*Versus*-Host Disease in Humans

**DOI:** 10.3389/fimmu.2021.700857

**Published:** 2021-09-03

**Authors:** Qingxiao Song, Xiaohui Kong, Paul J. Martin, Defu Zeng

**Affiliations:** ^1^Riggs Institute, The Beckman Research Institute, City of Hope National Medical Center, Duarte, CA, United States; ^2^Hematologic Malignancies and Stem Cell Transplantation Institute, City of Hope National Medical Center, Duarte, CA, United States; ^3^Fujian Medical University Center of Translational Hematology, Fujian Medical University Union Hospital, Fuzhou, China; ^4^Division of Clinical Research, Fred Hutchinson Cancer Research Center, Seattle, WA, United States; ^5^Department of Medicine, University of Washington, Seattle, WA, United States

**Keywords:** hematopoietic cell transplantation, chronic graft-*versus*-host disease, mouse models, tissue resident memory T cell, B cell

## Abstract

Allogeneic hematopoietic cell transplantation (allo-HCT) is a curative therapy for hematologic malignancies, but its success is complicated by graft-*versus*-host disease (GVHD). GVHD can be divided into acute and chronic types. Acute GVHD represents an acute alloimmune inflammatory response initiated by donor T cells that recognize recipient alloantigens. Chronic GVHD has a more complex pathophysiology involving donor-derived T cells that recognize recipient-specific antigens, donor-specific antigens, and antigens shared by the recipient and donor. Antibodies produced by donor B cells contribute to the pathogenesis of chronic GVHD but not acute GVHD. Acute GVHD can often be effectively controlled by treatment with corticosteroids or other immunosuppressant for a period of weeks, but successful control of chronic GVHD requires much longer treatment. Therefore, chronic GVHD remains the major cause of long-term morbidity and mortality after allo-HCT. Murine models of allo-HCT have made great contributions to our understanding pathogenesis of acute and chronic GVHD. In this review, we summarize new mechanistic findings from murine models of chronic GVHD, and we discuss the relevance of these insights to chronic GVHD pathogenesis in humans and their potential impact on clinical prevention and treatment.

## Introduction

Allogeneic hematopoietic cell transplantation (allo-HCT) offers a way to eliminate residual malignant cells and prevent relapse by taking advantage of the graft-*versus*-leukemia/lymphoma (GVL) activity of alloreactive donor T cells (1–6). However, the same alloreactive T cells also mediate graft-*versus*-host disease (GVHD) (7–9). Acute GVHD is an acute alloimmune inflammatory response characterized by infiltration of donor T cells that cause apoptosis and necroptosis of epithelial cells in GVHD target tissues (10–12). Chronic GVHD is an autoimmune-like chronic inflammation variably characterized with lymphopenia, IgG autoantibodies in the serum (13), moderate donor cell infiltration, and fibrosis in certain target tissues (14). Chronic GVHD often occurs as a sequel of acute GVHD, although chronic GVHD can occur in the absence of overt acute GVHD (15). In humans, acute and chronic GVHD can both involve the skin, liver, and gut, whereas prototypical target organs affected by chronic GVHD include salivary and lacrimal glands, oral mucosa, subcutaneous connective tissue and adipose tissue, lung, genital tract, and esophagus (15–18). Clinical manifestations of chronic GVHD typically begin between 2 and 12 months after allo-HCT (15, 19). In one retrospective study, 75% of the patients diagnosed with chronic GVHD had prior acute GVHD, and in 10% of the patients, acute GVHD evolved directly into chronic GVHD (20).

Studies of GVHD pathogenesis in humans are limited by the inaccessibility of target organ tissues other than the skin. Therefore, preclinical animal models represent important tools for elucidating the pathogenic processes leading to acute and chronic GVHD (21, 22). Murine models of allo-HCT have become the most important animal models for the GVHD mechanistic pathogenic studies, owing to the availability of genetically modified strains (21, 22), although work with canine and nonhuman primate models has produced important contributions (23, 24). Murine models of allo-HCT have demonstrated the role of recipient mismatching for major and minor histocompatibility antigens in triggering acute GVHD. These models have also elucidated the role of T-cell subsets and cytokines in acute GVHD pathogenesis (25–36). As one example, observations from murine models that NKT cells specific for nonpolymorphic CD1d suppressed acute GVHD (37) and preservation of NKT cells by conditioning regimens consisting of total lymphoid irradiation (TLI) and antithymocytes cell globulin (ATG) prevented GVHD while preserving GVL activity (38, 39) have been successfully translated into clinical application in humans (40, 41). Similarly, observations from murine models that removal of naïve T cells can ameliorate GVHD while preserving GVL activity (42, 43) have also been successfully translated into clinical application in humans (44).

Modeling chronic GVHD appeared to be more complicated, but murine models of chronic GVHD have evolved and improved during the past three decades. It was initially thought that murine models of autoimmune-like chronic GVHD required specific donor-recipient combinations that differ from those used to study acute GVHD (21, 22, 45). In this review, we will describe how we have used identical allogeneic donor and recipient strain combinations to induce acute GVHD mediated by alloreactive T cells and to induce autoimmune-like chronic GVHD. In these models, acute and chronic GVHD can occur sequentially in murine recipients (46), similar to what most often occurs in humans (15). These murine models also reflect the characteristic features of autoimmune-like chronic GVHD in humans (46). We will also summarize new insights into chronic GVHD pathogenesis through the murine models.

## A Murine Model Can Reflect Characteristic Features of Chronic GVHD in Patients

We recently found that induction of acute and chronic GVHD does not require different donor and host combination (46). With the commonly used acute GVHD model of C57BL/6 donor to MHC-mismatched BALB/c recipient, acute GVHD recipients develop into chronic GVHD as long as they survive for up to 60 days after allo-HCT (46). The induction of both acute and chronic GVHD can be achieved by adjusting donor T-cell numbers in the graft, and chronic GVHD in the absence of acute GVHD can be induced by injection of purified donor CD8^+^ T cells alone with T-cell–depleted bone marrow cells (46). Recipients with chronic GVHD induced by whole splenic T cells or by sorted donor CD8^+^ T cells both have lymphopenia, damage in the thymus, serum autoantibodies, and damage in small intestine, liver, lung, skin, and salivary and lacrimal glands, together with collagen deposition and fibrosis in target organ tissues (46, 47). The recipients clearly showed lymphocytic bronchiolitis and interstitial collagen deposition in the lung (46, 47), although bronchial obstruction (BO) observed in a murine model conditioned with TBI plus cyclophosphamide (CY) (48) was not observed in our models. BO in murine model of chronic GVHD may require special conditioning. In addition, as summarized in **Table 1**, chronic GVHD can be induced with low-dose splenic T cells in other MHC-mismatched or MHC-matched donor–recipient combinations, including MHC-mismatched C57BL/6 (H-2^b^) donor to B10BR (H-2^k^) recipient and MHC-matched LP/J (H-2^b^) donor to C57BL/6 (H-2^b^) recipient and DBA/2 (H-2^d^) or B10D2 (H-2^d^) donor to BALB/c (H-2^d^) recipient models (46, 49–55). Chronic GVHD with little acute GVHD can also be induced by naïve CD8^+^ T cells from C3H.SW (H-2D^b^, CD45.2) donor to MHC-matched B6/SJL (H-2D^b^, CD45.1) recipient (46, 56) or from C57BL/6 donor to MHC-mismatched BALB/c recipient models (46).

**Table 1:** Summary of murine models of cGVHD.

Donor strain	Recipient strain	Conditioning regiment	Genetics	Main cell type contributing to phenotype	Cell type and dose	Outcome	Reference
C57BL/6 (H-2^b^)	BALB/c (H-2^d^)	850 cGy	Mismatched for MHCI, MHCII, and miHAs	CD4^+^, CD8^+^ T, and B cells	2.5 × 10^6^ T-cell–depleted (TCD) BM cells and 0.5–1.25 × 10^6^ unfractionated spleen cells or 0.5 × 10^6^ CD4^+^ or 0.5–5 × 10^6^ CD8^+^ T cells	Systemic disease including (1) damages in the acute and chronic GVHD overlapping targets such as thymus, gut, liver, lung, and skin, as well as chronic GVHD prototypical targets salivary and lacrimal glands; (2) increased serum autoantibodies and tissue antibody deposition; (3) collagen deposition and fibrosis in target organ tissues.	Wu et al. (46) and Kong et al. (47)
C57BL/6 (H-2^b^)	B10BR (H-2^k^)	Cyclophosphamide-treated (120 mg/kg/day, days −3 and −2), irradiated (8.3 Gy by radiograph, day −1)	Mismatched for MHCI, MHCII, and miHAs	CD4^+^ and CD8^+^ T	TCD-BM and 0.75 × 10^5^ purified splenic T cells	Fibrosis with bronchiolitis obliterans	Katelyn Paz et al. (49)
LP/J (H-2^b^)	C57BL/6 (H-2^b^)	900–1,100 cGy	MHC-matched and miHA-mismatched	CD4^+^ and CD8^+^ T	Whole spleen (10 × 10^6^) and TCD-BM (2.5 × 10^6^)	Skin scleroderma	Deng et al. (50), Hamilton and Parkman (51), and DeClerck et al. (52)
DBA/2 (H-2^d^)	BALB/c (H-2^d^)	650 cGy	MHC-matched and miHA-mismatched	CD4^+^ T and B cells	2.5–10 × 10^7^ whole spleen cells	Autoantibodies; skin scleroderma; kidney damage	Zhang et al. (53) and Zhao et al. (54)
B10D2 (H-2^d^)	BALB/c (H-2^d^)	850 cGy	MHC-matched and miHA-mismatched	CD4^+^ and CD8^+^ T	Whole spleen (10 × 10^6^) and TCD-BM (2.5 × 10^6^)	Skin scleroderma? Systemic disease?	Deng et al. (50), Korngold and Sprent (27), and Eyrich et al. (55)
C3H.SW (H-2D^b^, CD45.2)	C57BL/6SJL (B6/SJL, H-2D^b^, CD45.1)	1,000 cGy	MHC-matched and miHA-mismatched	Naïve CD8^+^ T	TCD-BM (5 × 10^6^) and CD44^low^CD8^+^ T cells (2 × 10^6^)	Systemic disease including thymus, skin, liver, and gastrointestinal tract damage.	Zhang et al. (56)

Many characteristic features of acute and chronic GVHD in humans can be reflected by any murine model, although no single murine model captures the entire spectrum of abnormalities observed in humans, just as no single patient can represent the full spectrum of abnormalities that can be caused by the disease. Establishing murine models of acute and chronic GVHD does not require any specific donor and recipient combination. The key is to adjust the number of donor T cells in the graft to allow the recipients to survive acute phase, such that inflammation in acute GVHD can evolve into the myriad features unique to chronic GVHD.

## Autoreactive Pathogenic CD4^+^ T Cells in Chronic GVHD Are Derived From Both Mature CD4^+^ T Cells in the Graft and *De Novo*-Generated CD4^+^ T Cells in the Thymus

The diversity of TCRs are randomly generated by VDJ recombination during positive selection in the thymus, and autoreactive T cells in healthy thymus are depleted by negative selection in the thymic medullary mediated by medullary epithelial cells (mTECs) and dendritic cells (DCs). The mTECs express tissue-restricted antigens (TRA) in AIRE- or Fezf2-dependent manner (57–59). The CD11c^+^ DCs in the thymic medullary include CD11c^+^B220^+^PDCA-1^+^ plasmacytoid DCs (pDCs), CD8^+^SIRPα^−^ thymus-resident DCs (tDCs), and CD8^−^SIRPα^+^ migratory DCs (mDCs) (60–62). TRA from mTECs can be picked up by thymic DCs, and TRA from periphery tissues can be brought into the thymus by mDCs (59). pDCs and tDCs augment thymic negative selection with limited impact on Treg generation; in contrast, mDCs augment both negative selection and tTreg generation in the thymus (60–63). The mTEC- and DC-mediated negative selection deletes most of the autoreactive thymocytes; however, the deletion is not 100%, and a small portion of the autoreactive T cells is exported to the periphery (64). The residual autoreactive T cells in the periphery of healthy individuals are well regulated and controlled by peripheral tolerance mechanisms consisting of regulatory T cells and tolerogenic DCs (65).

**Figure 1:**
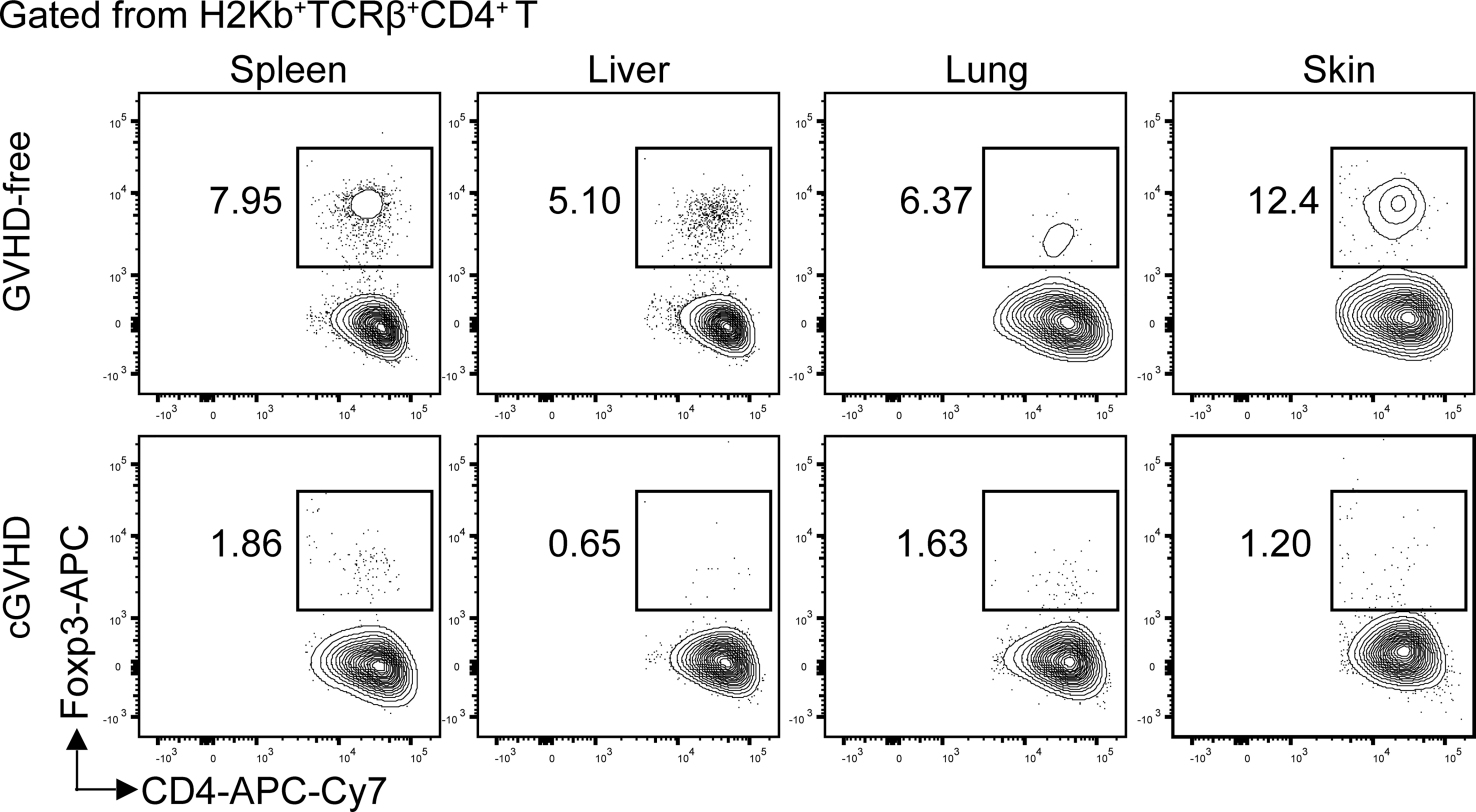
Loss of Foxp3^+^CD4^+^ Treg cells in the target tissues of chronic GVHD recipients. Lethal TBI-conditioned BALB/c recipients were given T-cell–depleted bone marrow cells (TCD-BM, 2.5 × 10^6^) only from C57BL/6 donors as GVHD-free control or given TCD-BM plus spleen cells (1 × 10^6^) for induction of chronic GVHD. Sixty days after HCT, the spleen, liver, lung, and skin tissue mononuclear cells were stained with anti-H-2K^b^, TCRb, CD4, and FoxP3. The gated donor-type H-2K^b+^CD4^+^TCRβ^+^ T cells are shown in CD4 *versus* Foxp3. The FoxP3^+^ Treg cells are boxed, and the percentage of the Treg cells among the CD4^+^ T cells is shown beside the box. One representative is shown of four recipients in each group.

Residual autoreactive T cells in the graft from healthy donors are expanded after allo-HCT due to breakdown of tolerance mechanisms. In murine models, as depicted in **Figure 1**, early after allo-HCT, donor T cells including the residual autoreactive T cells in the graft are activated by host-type APCs and differentiate into Th/Tc1 cells, and they infiltrate GVHD target tissues including gut, liver, lung, skin, thymus, and bone marrow to mediate acute GVHD. Autoreactive CD4^+^ T cells express promiscuous TCRs that cross-react with both self-MHC-antigen complex and allo-MHC-antigen complex (66). Since autoimmune-like chronic GVHD can be induced in thymectomized and athymic recipients (53), the autoreactive CD4^+^ T cells in those recipients are most likely derived from the residual autoreactive CD4^+^ T cells in the graft that expanded during alloimmune responses (53, 54). The autoreactive CD4^+^ T cells recognize both donor antigen-MHC complex and host antigen-MHC complex, such that they first act as alloreactive T cells and are activated by host-type APCs, and then they act as autoreactive T cells and are expanded by donor-type APCs, particularly by the activated donor-type B cells presenting donor- or host-type antigens (67).

The autoreactive CD4^+^ T cells in chronic GVHD recipients are also derived from *de novo*-generated CD4^+^ T cells from GVHD-damaged thymus (46). The thymus of allo-HCT recipients can be damaged by condition regimen and GVHD. The thymus damage by conditioning regimen alone can recover in an IL-22-dependent manner (68). Alloreactive CD4^+^ T and CD8^+^ T cells mediate damage of mTECs that mediate negative selection of autoreactive T cells (34, 46, 69). Although donor-type DCs augment negative selection of autoreactive antidonor and antihost T cells in non-GVHD recipients with mixed or complete chimerism (70, 71), donor-type DCs no longer augment negative selection of the autoreactive T cells in GVHD recipients due to loss or dysfunction of donor-type DCs (46, 72). Therefore, damage of thymus, especially by GVHD leads to an increased generation of autoreactive T cells.

Autoreactive CD4^+^ T cells in chronic GVHD recipients include those derived from the mature T cells in the graft or those from *de novo*-generation in the damaged thymus. In the recipients with overt acute and chronic GVHD, majority of pathogenic CD4^+^ T cells are from donor-type CD4^+^ T cells from the graft (46, 47). This may result from rapid destruction of thymus by acute GVHD that ends the thymic production. However, in the recipients transplanted with sorted CD8^+^ T cells and that developed little acute GVHD, *de novo*-generated donor-type CD4^+^ T cells are required for induction of chronic GVHD (46). The autoreactive CD4^+^ T cells from both sources recognize donor antigen-MHC complex and host antigen-MHC complex, and they interact with autoreactive B cells to produce autoantibodies that further damaged the thymus and causes lymphopenia in chronic GVHD recipients (46, 50, 67, 73). Therefore, the autoreactive CD4^+^ T cells derived from the preexisting autoreactive CD4^+^ T cells in the graft play a major role in mediating chronic GVHD pathogenesis in recipients with overt acute and chronic GVHD, and the *de novo*-generated autoreactive CD4^+^ T cells from damaged thymus play a major role in chronic GVHD pathogenesis in recipients with little prior acute GVHD.

## Chronic GVHD Pathogenesis Does Not Require Germinal Centers and Its Onset Is Associated With Destruction of Lympho-Follicles and Germinal Centers

Patients with active chronic GVHD have marked reduction of PD-1^hi^CXCR5^+^CD4^+^ follicular T helper cells (Tfh) among peripheral blood mononuclear cells (PBMC), but high serum concentrations of IgG autoantibodies and CXCL13, the ligand of CXCR5 (13, 74), suggesting intense T helper activity for B cells. The results were interpreted to indicate that Tfh had been recruited into germinal centers of lymphoid follicles in secondary lymphoid organs, consistent with previous preclinical studies showing that chronic GVHD onset was associated with enlarged germinal centers in some murine models of chronic GVHD (74, 75). However, this interpretation conflict with observations that patients with chronic GVHD usually have lymphopenia (76–79), and that somatic hypermutation (SHM) in the memory B cells is low at 1 year after HCT (80, 81).

SHM takes place in the B cells during differentiation in the GCs (82–84). With variety murine models, we have demonstrated that chronic GVHD onset is associated with destruction of lymphoid follicles and GCs in the spleen. In addition, we showed that GC formation is not required for induction of chronic GVHD, because recipients with an absence of BCL6 in donor B cells that could not form GCs nonetheless developed chronic GVHD (50, 73). Recipients with overt chronic GVHD had no detectable GCs, Tfh cells, or GC B cells, although recipients with mild chronic GVHD had remnants of GCs, residual Tfh, and GC B cells (47, 50, 73).

## Extrafollicular PSGL1^lo^CD4^+^ T and B Cell Interactions Augment Autoimmune Development and Chronic GVHD Pathogenesis

P-selectin glycoprotein ligand 1 (PSGL1, also known as CD162) is an adhesion and coinhibitory receptor; PSGL1 are widely expressed in almost all T cells in the blood and binds to E-selectin and P-selectin (85, 86). A subset of activated CD4^+^ T cells in the spleen of SLE mice downregulate expression of PSGL1 and become CD44^hi^CD62L^-^PSGL1^lo^CD4^+^ T (PSGL1^lo^CD4^+^ T) cells (87). PSGL1^lo^CD4^+^ T cells localize at the extrafollicular sites of systemic lupus mice and express high levels of CXCR4, ICOS, and CD40L without expression of CXCR5 (88). We served that chronic GVHD onset is associated with expansion of PSGL1^lo^CD4^+^ T helpers in the GVHD target tissues (47, 50). Extrafollicular PSGL1^lo^CD4^+^ T helpers for autoreactive B cells were first identified as CD4^+^ T helpers in the spleen of systemic lupus mice (87). The differentiation of the PSGL1^lo^CD4^+^ T helpers in chronic GVHD recipients depends on the IL-6R-Stat3-BCL6 pathway, and Stat3 or BCL6 deficiency in donor CD4^+^ T cells prevented expansion of the PSGL1^lo^CD4^+^ T cells in GVHD target tissues (47, 50). The PSGL1^lo^CD4^+^ T cell interaction with B cells results in autoantibody production and augmented thymus damage early after HCT (50). Prevention of PSGL1^lo^CD4^+^ T expansion by BCL6 or Stat3 deficiency and by blockade of ICOS or PD-1 interaction with ICOSL or PD-L2 on B cells markedly reduced serum concentrations of autoantibodies and decreased the severity of chronic GVHD (47, 50). In addition, we observed that chronic GVHD tissues had high levels of CXCL13 as measured with liver tissue homogenates, and PSGL1^lo^CD4^+^ T cells expressed high levels of CXCL13 mRNA (Kong, unpublished data). Taken together, these results suggest that the low number of Tfh cells in the PBMC of active chronic GVHD patients is unlikely due to redistribution of the Tfh cells into GCs in the lymphoid follicles, and it is more likely due to the destruction of GCs and lymphoid follicles. The high concentrations of CXCL13 and IgG autoantibodies in the serum of the patients may result from expansion of extrafollicular CD4^+^ T and B cells in GVHD target tissues.

## Extrafollicular PSGL1^lo^CD4^+^ T Helper Cells Are Tissue Resident Memory T Cells That Interact With Memory B Cells in the GVHD Target Tissues During Chronic GVHD Pathogenesis

As mentioned above, extrafollicular PSGL1^lo^CD4^+^ T cells were identified in the spleen of systemic lupus more than a decade ago (87), but their role in human systemic lupus pathogenesis remains unknown. We have recently found that PSGL1^lo^CD4^+^ T cells were not detectable in the peripheral blood of murine or human chronic GVHD recipients (47). Instead, they were CD4^+^ tissue-resident memory T (Trm) cells with upregulated expression of CD69, CXCR6, P2RX7, and PD-1 and downregulated expression of Klf2, S1PR1, and CCR7 (47), consistent with Trm cell phenotype reported by others in infection and autoimmune colitis models (89). These observations explain why extrafollicular PSGL1^lo^CD4^+^ T cells are not detectable in the peripheral blood of mice or patients with chronic GVHD. This may also explain why their role in the pathogenesis of systemic lupus has not been investigated in humans.

The PSGL1^lo^CD4^+^ Trm cells interact with memory B cells in the GVHD target tissues in murine recipients, humanized murine recipients, and in the liver of cGVHD patients (47). The humanized murine model was established by injection of HLA-A2^−^DR4^−^ human PBMC into MHC^−/−^HLA-A2^+^DR4^+^ NSG mice (47). The PSGL1^lo^CD4^+^ T cells were juxtaposed to memory B cells in the liver of murine recipients, humanized murine recipients, and patients with chronic GVHD, as indicated by immunofluorescent and immunohistochemistry staining of the tissue-infiltrating cells (47). Sorted PSGL1^lo^CD4^+^ T cells from GVHD target tissues (liver and lung) of murine and humanized murine recipients augmented *in vitro* differentiation of syngeneic or autologous memory B cells but not naïve B cells into IgG-producing plasma cells in a manner that depended on PD-1/PD-L2 interaction and IL-21 (47).

On the other hand, the expansion of human memory B and plasma B cells in the GVHD target tissue liver and lung of humanized murine recipients was associated with expansion of PSGL1^lo^CD4^+^ T cells, but little B cell activation and expansion were observed in the MHC^−/−^ control recipients (47). We also observed that PD-1 deficiency in donor T cells and PD-L2 deficiency in donor B cells were associated with reduction of serum anti-dsDNA, reduction of tissue IgG deposition, reduction of tissue fibrosis, and reduction of chronic GVHD severity (47). Finally, sorted PD-1^+/+^ or PD-1^−/−^ PSGL1^lo^CD4^+^ T and PSGL1^hi^CD4^+^ T cells from liver and lung GVHD target tissues were adoptively transferred into GVHD-free adoptive recipients with PD-L2^+/+^ or PD-L2^−/−^ B cells. The results showed that PSGL1^lo^ and PSGL1^hi^ CD4^+^ memory T cells preferentially migrated back to the original GVHD target tissues in the adoptive recipients, but only PSGL1^lo^CD4^+^ T cells augmented expansion of plasma cells in the GVHD target tissues and increased serum concentration of total IgG and anti-dsDNA-IgG in a manner that required PD-1 interaction with PD-L2 (47). Taken together, these results show that PSGL1^lo^CD4^+^ Trm cell interaction with memory B cells in GVHD target tissues contributes to perpetuation of chronic GVHD pathogenesis.

## Extrafollicular PSGL1^lo^CD4^+^ T Helpers Are Derived From PSGL1^hi^CD4^+^ T Cells in the Graft in an IL-6-Stat3-BCL6-Dependent Manner

We observed that all peripheral blood CD4^+^ T cells from healthy murine and human donors were PSGL1^hi^ (47). After transplantation into murine and humanized murine recipients, a portion (5%–20%) of PSGL1^hi^CD4^+^ T cells differentiated into PSGL1^lo^CD4^+^ Trm cells in an IL-6-Stat3-dependent manner (47) because Stat3 deficiency in the CD4^+^ T cells and administration of blocking anti-IL-6R mAb markedly reduced the expansion of PSGL1^lo^CD4^+^ T cells in the GVHD target tissues of murine recipients (47). We have also observed expansion of *de novo*-generated PSGL1^lo^CD4^+^ T cells in chronic GVHD recipients induced by sorted donor CD8^+^ T cells (Kong, unpublished data). These results indicate that PSGL1^lo^CD4^+^ T differentiation is similar to prefollicular CD4^+^ T differentiation that is controlled by IL-6-Stat3-BCL6 pathways (50, 84).

## Circulating Antibodies Augment Sclerodermatous Cutaneous Chronic GVHD

In humans, autoantibodies such as PDGF-1 have been associated with increased severity of cutaneous chronic GVHD (90). We found that high serum concentrations of autoantibody were associated increased IgG deposition and fibrosis in the skin tissues of murine and humanized murine recipients (47). Donor-derived IgG antibodies were required to perpetuate cutaneous chronic GVHD (73). Unexpectedly, we found no PSGL1^lo^CD4^+^ T or B cells in the skin tissues of murine or humanized murine recipients with chronic GVHD, although PSGL1^lo^CD4^+^ T and memory B cells were present in the liver and lung (47). Studies are in progress to determine whether B cells or PSGL1^lo^CD4^+^ T cells are present in the skin of patients with cutaneous chronic GVHD.

Taken together, the preclinical results indicate that circulating autoantibodies contribute to pathogenesis of cutaneous chronic GVHD. We also observed that circulating IgG antibodies augmented DC secretion of IL-23 and expansion of Th17 cells in the skin of chronic GVHD mice (73). MacDonald et al. showed that in an IL-17-dependent cutaneous chronic GVHD model, donor-type F4/80^+^CSF-1R^+^ type 2 macrophages augmented cutaneous chronic GVHD in a G-CSF but not GM-CSF-dependent manner, in which the macrophages mediate fibrosis *via* their production of TGF-β (91). Whether circulating IgG autoantibodies regulate the differentiation and expansion of type 2 macrophages during cutaneous GVHD remains to be studied.

## Loss of Functional Thymic DCs and Peripheral PD-L1^hi^ Plasmacytoid DCs May Contribute to Loss of Foxp3^+^CD4^+^ Treg Cells in Chronic GVHD Target Tissues

Chronic GVHD patients had markedly low percentages of Foxp3^+^CD4^+^ regulatory T (Treg) cells in the blood (92). Low-dose IL-2 preferentially expanded CD4^+^ Treg cells by binding to high affinity IL-2Rα (CD25) and ameliorated clinical manifestation of chronic GVHD (93–95). Consistently, in a chronic GVHD model with DBA/2 donors and BALB/c recipients, loss of CD4^+^ Treg cells was associated with chronic GVHD onset, and infusion of donor-type Treg cells prevented the disease onset or ameliorated the progression of chronic GVHD (96, 97). Importantly, we observed that percentages of Treg cells were high among CD4^+^ T cells in the spleen, liver, lung, and skin of healthy donor or GVHD-free recipients, but few Treg cells were found among CD4^+^ T cells in the same tissues from mice with chronic GVHD (**Figure 1**).

The low number of CD4^+^ Treg cells may result from reduced thymic Treg (tTreg) output, reduced differentiation of conventional CD4^+^ T (Tcon) cells into peripheral Treg (pTreg) cells, and reduced Treg expansion and survival in the periphery. Thymic damage or engraftment with MHCII^−/−^ donor DCs resulted in reduced generation of tTreg cells in GVHD recipients (98, 99), while engraftment of donor-type DCs increased donor- and host-type thymic tTreg generation in GVHD-free MHC-mismatched or haploidentical mixed chimeras (71, 100, 101). Plasmacytoid DCs that express high levels of PD-L1 (PD-L1^hi^ pDCs) augment Tcon differentiation into pTreg cells in a PD-L1/PD-1 interaction-dependent manner (102–104). DC PD-L1 interaction with CD80 on Treg cells also augments Treg survival and expansion (96). GVHD in bone marrow reduced the production of PD-L1^+^ pDCs, leading to reduced generation and expansion of Treg cells (105). Therefore, loss of functional DCs in the thymus and loss of bone marrow generation of PD-L1^hi^ pDCs may contribute to the marked reduction of Treg cells in the chronic GVHD recipients, and prevention of thymus and bone marrow GVHD as well as restoration of bone marrow production of pDCs might reverse chronic GVHD.

## Conclusions

In summary, with murine models of chronic GVHD, we have found that extrafollicular CD4^+^ T and B interactions and CD4^+^ Trm cells in the GVHD target tissues play critical roles in chronic GVHD pathogenesis, and these findings have been linked to chronic GVHD pathogenesis in humans through studies with humanized MHC^−/−^HLA-A2^+^DR4^+^ NSG mice and patient GVHD target tissues (47). These studies have provided new insights into chronic GVHD pathogenesis in humans.

**Figure 2:**
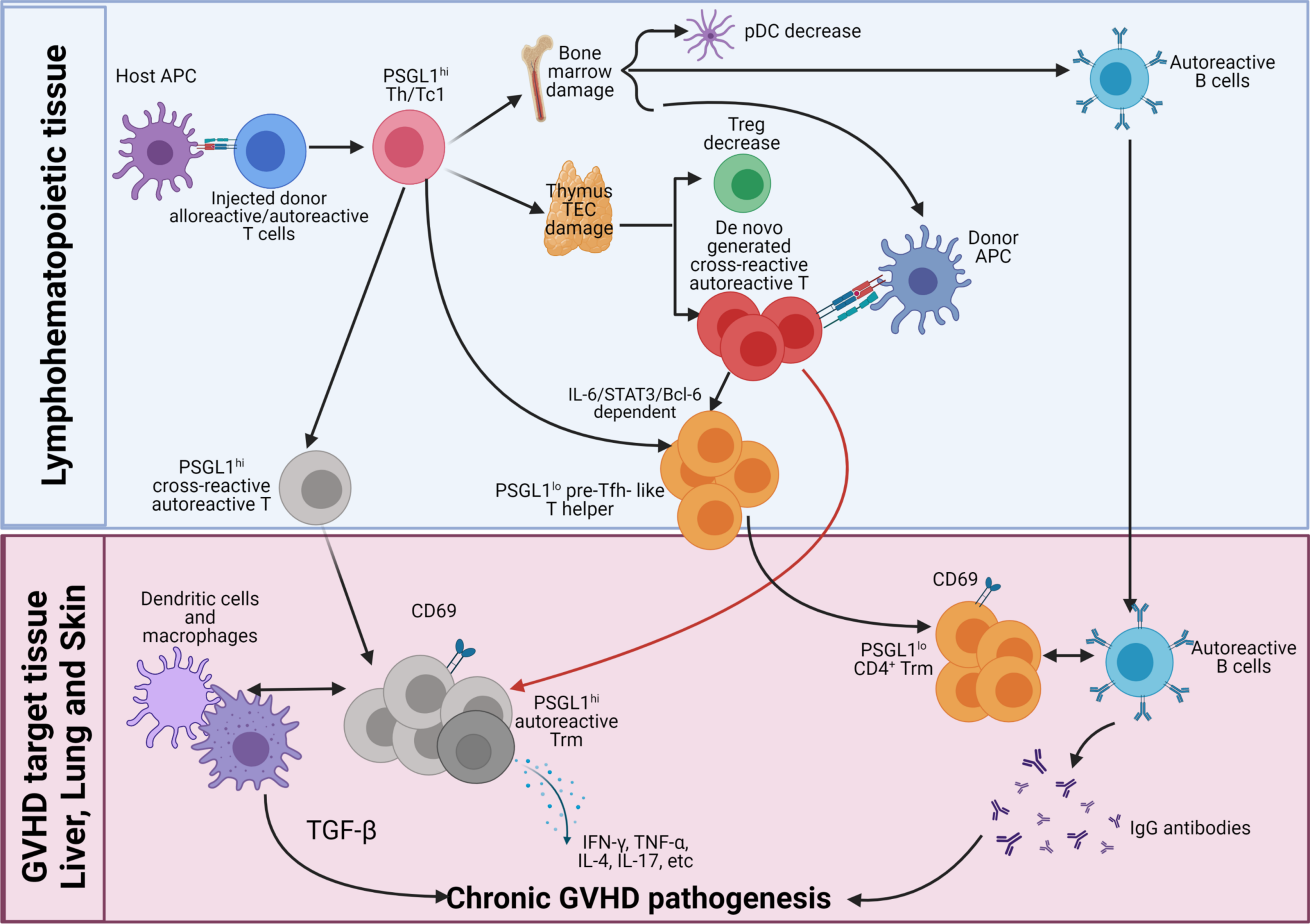
Pathogenesis of chronic GVHD. Early after allo-HCT, donor-type CD4^+^, and CD8^+^ T cells including autoreactive CD4^+^ T cells are activated by host APCs in the lymphoid tissues. The majority of the injected alloreactive T cells differentiate into PSGL1^hi^ Th1/Tc1 cells to cause acute GVHD. At the same time, some of the autoreactive CD4^+^ T cells differentiate into PSGL1^lo^CD4^+^ pre-Tfh-like cells *via* IL-6-Stat3-BCL6 pathway, and they interact with activated donor B cells to augment antibody production, and some of them remain PSGL1^hi^. The Th1/Tc1 cells infiltrate GVHD target tissues including thymus and bone marrow. Damage of thymic medullary epithelial cells (mTECs) leads to decreased generation of thymic Tregs (tTreg) cells and increased release of autoreactive T cells that are cross-reactive with donor antigen-MHC complex and host antigen-MHC complex. Damage of bone marrow microenvironment results in increased production of autoreactive B cells and reduced production of tolerogenic plasmacytoid dendritic cells (pDCs). Acute GVHD destroys lymphoid tissues. As acute GVHD subside into chronic GVHD, alloreactive pathogenic memory T, especially CD4^+^ memory T cells, that can cross-react with donor APCs become autoreactive CD4^+^ T cells and gather in the GVHD target tissue. The *de novo*-generated autoreactive CD4^+^ T cells from damaged thymus also infiltrate the GVHD target tissues. The autoreactive CD4^+^ T cells from both sources interact with donor-type APCs and become CD69^+^ tissue resident memory T (Trm) cells in the tissues. The PSGL1^hi^ autoreactive Trm cells interact with DCs and macrophages to mediate pathogenesis *via* their production of cytokines such as TGF-β, IFN-γ, TNF-α, IL-4, IL-17, and IL-22. The pre-Tfh-like PSGL1^lo^CD4^+^ helper T cells interact with B cells to augment memory B-cell differentiation into plasma cells that produce IgG autoantibodies. IgG autoantibodies enter circulation and deposit in the GVHD target tissues such as skin to augment GVHD pathogenesis.

As depicted in the diagram (**Figure 2**), we propose how donor CD4^+^ T cells mediate autoimmune-like chronic GVHD pathogenesis. Step 1, early after allo-HCT, in the lymphoid tissues, donor CD4^+^ and CD8^+^ T cells including cross-reactive residual autoreactive CD4^+^ T cells in the graft act as alloreactive CD4^+^ and CD8^+^ T cells; they are activated by interaction with host-type APCs. The alloreactive T cells differentiate into PSGL1^hi^ Th1/Tc1 cells and infiltrating GVHD target tissues. The small portion of autoreactive CD4^+^ T cells differentiate into PSGL1^hi^CD4^+^ memory T cells and PSGL1^lo^CD4^+^ pre-Tfh-like cells *via* IL-6-Stat3-BCL6 pathway. The alloreactive Th1/Tc1 cells migrate into and cause damage in the thymus and bone marrow, among other GVHD target tissues such as the liver, lung, and skin. Those alloreactive Th1/Tc1 cells also destroy secondary lymphoid tissues as time goes on. Due to GVHD damage of thymic medullary epithelial cells and defective negative selection, the thymus increases production of autoreactive CD4^+^ T cells that recognize both donor antigen-MHC complex and host antigen-MHC complex, as well as reduces production of CD4^+^ tTreg cells. Those cross-reactive autoreactive CD4^+^ T cells are activated in the periphery and infiltrate GVHD target tissues. The GVHD-damaged bone marrow has low production of tolerogenic PD-L1^hi^ pDCs, defective negative selection of autoreactive B cells, and markedly reduced output of B cells and myeloid cells, leading to lymphopenia with relative expansion of autoreactive B cells in the periphery.

Step 2, the cross-reactive autoreactive CD4^+^ T cells derived from the residual autoreactive CD4^+^ T cells in the graft and from *de novo*-generation in the damaged thymus interact with donor-type DCs/macrophages or B cells, leading to their survival and expansion after acute GVHD subsides. The cross-reactive autoreactive CD4^+^ T cells infiltrate GVHD target tissues and become CD69^+^ Trm cells. The PSGL1^hi^ Th1, Th2, and Th17 cross-reactive autoreactive Trm cells interact with DCs and macrophages to mediate chronic GVHD pathogenesis *via* their production of cytokines such as TGF-β, IFN-γ, TNF-α, IL-4, IL-17, and IL-22. The pre-Tfh-like autoreactive PSGL1^lo^CD4^+^ T cells become extrafollicular PSGL1^lo^CD4^+^ helper T cells in the GVHD target tissues (i.e., liver and lung). They attract and interact with autoreactive B cells in the tissues and become Trm cells. Their interaction with B cells augments memory B-cell differentiation into plasma cells that produce IgG autoantibodies that augment local inflammation and fibrosis or enter circulation. The circulating IgG antibodies deposit in the tissues such as skin to augment GVHD pathogenesis.

Finally, lack of tolerogenic pDCs and Treg cells allow the cross-reactive autoreactive CD4^+^ Trm cells that recognize both donor antigen-MHC complex and host antigen-MHC complex to continuously interact with DCs, macrophages, and B cells to perpetuate chronic GVHD pathogenesis. Therefore, PSGL1^hi^ and PSGL1^lo^ CD4^+^ Trm cells, macrophage, dendritic cells, B cells, and circulating IgG autoantibodies, all contribute to the pathogenesis of chronic GVHD, but CD4^+^ Trm cells play the essential role. Targeting autoreactive CD4^+^ Trm cells in the GVHD target tissues for treatment of chronic GVHD is under investigation.

We would like to point out that the proposed model of cGVHD pathogenesis is more relevant to chronic GVHD pathogenesis in recipients with obvious acute GVHD. However, in the clinic, some chronic GVHD patients did not have a clear phase of acute GVHD. The origin of the pathogenic T cells in those patients remains unclear. They may derive from *de novo* thymus-generated T cells because our murine model showed that sorted donor CD8^+^ T cells induced thymus damage and chronic GVHD in the absence of acute GVHD (46). The roles of Tfh and extrafollicular PSGL1^lo^CD4^+^ T-cell interaction with B cells in the pathogenesis of chronic GVHD without obvious acute GVHD remain unclear and are under investigation.

## Author Contributions

QS, XK, and DZ wrote the review manuscript. PM critically reviewed and edited the manuscript. All authors contributed to the article and approved the submitted version.

## Funding

This work was supported by the National Institutes of Health Grant R01 AI066008 and R01 CA228465 (to DZ).

## Conflict of Interest

The authors declare that the research was conducted in the absence of any commercial or financial relationships that could be construed as a potential conflict of interest.

## Publisher’s Note

All claims expressed in this article are solely those of the authors and do not necessarily represent those of their affiliated organizations, or those of the publisher, the editors and the reviewers. Any product that may be evaluated in this article, or claim that may be made by its manufacturer, is not guaranteed or endorsed by the publisher.
